# Early Structural and Vascular Changes after Within-24 Hours Vitrectomy for Recent Onset Rhegmatogenous Retinal Detachment Treatment: A Pilot Study Comparing Bisected Macula and Not Bisected Macula

**DOI:** 10.3390/jcm11123498

**Published:** 2022-06-17

**Authors:** Rossella D’Aloisio, Matteo Gironi, Tommaso Verdina, Chiara Vivarelli, Riccardo Leonelli, Cesare Mariotti, Shaniko Kaleci, Lisa Toto, Rodolfo Mastropasqua

**Affiliations:** 1 Ophthalmology Clinic, Department of Medicine and Science of Ageing, University Gabriele D’Annunzio Chieti-Pescara, 66100 Chieti, Italy; l.toto@unich.it (L.T.); rodolfo.mastropasqua@gmail.com (R.M.); 2 Ophthalmology Clinic, Azienda Ospedaliero-Universitaria di Modena, University of Modena and Reggio Emilia, 41122 Modena, Italy; matteo.gironi@hotmail.it (M.G.); tommaso.verdina@gmail.com (T.V.); chia.vivarelli@gmail.com (C.V.); leonelliriccardo@outlook.it (R.L.); 3 Eye Clinic, AOU Ospedali Riuniti Ancona-Polytechnic University of Marche, 60121 Ancona, Italy; cesare.mariotti@ospedaliriuniti.marche.it; 4 Department of Surgical, Medical, Dental and Morphological Sciences with Interest Transplant, Oncological and Regenerative Medicine, Azienda Ospedaliero-Universitaria di Modena, University of Modena and Reggio Emilia, 41122 Modena, Italy; shaniko.kaleci@unimore.it

**Keywords:** rhegmatogenous retinal detachment, optical coherence tomography angiography, vitrectomy, foveal avascular zone, macular vessel density

## Abstract

Background: In this study we aimed at investigating macular perfusion/anatomical changes in eyes with early onset rhegmatogenous retinal detachment (RRD) after prompt surgery within 24 hours, comparing a bisected macula and not bisected macula RRD. Methods: In this prospective observational study, 14 eyes of 14 patients who underwent within-24 hours vitreoretinal surgery for early onset RRD were enrolled. Patients were further divided into two subgroups: the not bisected macula group (NBM group) and the bisected macula group (BM group). At baseline and 3-month follow up, macular architecture and vessel analysis were assessed using optical coherence tomography angiography (OCTA) imaging. In detail, quantitative and qualitative analyses of the macular area were performed to quantify topographical retinal perfusion changes after surgery, calculating the foveal avascular zone (FAZ), vessel density (VD) and vessel length density (VLD) at the superficial capillary plexus (SCP) and deep capillary plexus (DCP). Results: Most cases (43%) were superotemporal RRD. Primary retinal reattachment was obtained in all cases, without recurrences within 3-month follow up. After surgery, a significant FAZ enlargement was observed at both the SCP and DCP level (*p* < 0.001; *p* < 0.05), with a significant effect of time noted between the two time points in the NBM and BM subanalysis (F = 3.68; *p* < 0.017). An excellent functional outcome was maintained for the whole follow-up. On the other hand, after surgery, perfusion parameters did not change significantly apart from the vessel density of the inferior macular sector at the DCP level (*p* = 0.03). Conclusions: Our findings suggest that the macular perfusion of eyes with RRD is still preserved if the surgery is performed really promptly, thus highlighting the great importance of a correct timing for surgery. OCTA analysis allows for a better understanding of the pathophysiological mechanisms underneath early vascular microarchitecture modifications of the posterior pole in retinal detachment, differentiating the two types of RRD not completely involving the fovea (BM and NBM).

## 1. Introduction

Primary rhegmatogenous retinal detachment (RRD) is an acute threat to visual impairment due to a retinal break that allows the passage of vitreous fluid into the subretinal space. The result of this event is the separation of the neurosensory retina from the retinal pigment epithelium (RPE), requiring early surgical management [1]. Considering the morphology of the RRD, a high anatomical success rate of 82–95% has been detected following appropriate surgery [2,3,4,5,6,7]. However, the anatomical success is not always linked to functional visual recovery and involvement of the macula in the retinal detachment pathogenesis is one of the most important prognostic factors for visual prognosis [8]. In clinical practice the combination of optical coherence tomography (OCT) and optical coherence tomography angiography (OCTA) has allowed a more detailed study of some suboptimal functional recovery causes after surgery, including a refractory cystoid macular edema (CME), persistent subretinal fluid (SRF) and epiretinal membrane (ERM) combined with alteration of the inner segment/outer segment (IS/OS) [9,10,11,12,13], and a more accurate analysis of the pathophysiological changes occurring in the macular microcirculation in retinal diseases, such as diabetic retinopathy, age-related macular degeneration, retinal vein occlusions, uveitis and macular telangiectasias [14]. Several studies have investigated retinal microvasculature and its changes in ocular diseases after vitreoretinal surgery. Some studies based on fluorescein angiography, found a lower retinal circulation time in the detached retina and an increase in vascular resistance, leading to tissue hypoxia and to the release of inflammatory mediators [15,16]. Afterwards, some OCTA-based studies reported an enlargement of the FAZ area and a decrease in retinal vessel density, after vitrectomy, in macula-OFF RRD in comparison to eyes with Macula-ON RRD and fellow eyes [17,18,19]. Some prospective studies on perfusion and anatomical macular changes after surgery provided controversial results depending on surgery timing and retinal detachment features. 

In this study we aimed at investigating macular perfusion/architecture modifications in eyes with early onset rhegmatogenous retinal detachment not completely involving the fovea, comparing bisected macula (BM) and not bisected macula (NBM) detachment. We aimed at highlighting the importance of a prompt surgery for visual acuity and macular perfusion status preservation, as well.

## 2. Materials and Methods

This study was reviewed and approved by the Local Ethics Committee of the University of Modena (Prot. AOU 0029636/20; date 20 October 2020) and Reggio Emilia and was conducted in accordance with the ethical standards of Declaration of Helsinki. 

### 2.1. Study Subjects

In this prospective observational study, 14 eyes of 14 patients, who underwent vitreoretinal surgery for RRD, were enrolled between November 2020 and April 2021 at the Department of Ophthalmology of University of Modena and Reggio Emilia, Italy. 

Only patients who successfully underwent a single uncomplicated vitreoretinal surgery for primary, recent onset (<24 h), macula-ON RRD were included. Exclusion criteria for the study were: (a) history of eye surgery within 6 previous months, (b) retinal vascular diseases, glaucoma and any other ocular diseases that may affect visual acuity or retinal/choriocapillary perfusion, (c) highly myopic eyes with axial length > 26.5 mm, (d) poor collaboration of patients during visits. All fellow eyes were healthy at the moment of data recording, and were considered as controls. 

For all patients, a complete ophthalmic evaluation, including best-corrected visual acuity (BCVA, Snellen’s chart, reported in LogMAR scale), slit-lamp examination, lens status (according to Lens Opacities Classification System III), applanation tonometry, axial length (AL, Aladdin TOPCON, noncontact optical low-coherence interferometry), swept source (SD)-OCT and OCTA acquisition, was performed at baseline and follow-up visits in both eyes. Postoperative data collection was set at 3 months follow-up, after complete intraocular gas reabsorption and media opacity resolutions. 

Patients were further divided into two subgroups: not bisected macula group (NBM group), with retina completely attached in the area subtended by 2.5 mm diameter circle around foveola, and bisected macula group (BM group), with subretinal fluid present in that area but without causing a complete lifting. The bisected macula RD has been considered to be a macula partially involving the fovea but not completely. Extension of detachment was recorded by preoperative schematic drawing. The retina was divided into 4 quadrants of 90 degrees amplitude (superior: S, nasal: N, inferior: I, temporal: T, as reported in Table 1). A quadrant was considered involved in the detachment if it included at least 1/3 of the area.

### 2.2. OCTA Imaging

The OCT and OCTA images were acquired using Canon OCT HS100 angiography^®^ (Canon Inc., Tokyo, Japan), whose software aims, using an appropriate algorithm, to generate a volumetric rendering of the blood flow from the internal limiting membrane (ILM) to the choroid and to allow direct visualization of the macular microcirculation. The machine performs 70,000 scans/s and the segmentation of the retinal layers is automatic and performed by the software (RX Capture for OCT-HS100^®^) to generate front projection images of the superficial capillary plexus (SCP) and the deep capillary plexus (DCP).

Poor-quality images (signal strength index < 8) with either significant motion artifact or extensive incorrect segmentation were excluded and repeated.

The superficial capillary plexus was analyzed considering the macular retinal section ranging from 3 µm under the ILM up to 15 µm under the internal plexiform layer (IPL). The deep capillary plexus was the thickness between 15 and 70 µm below the IPL.

Centered on the fovea, 3 × 3 mm OCTA scans were performed in both eyes. Image review was performed by two vitreoretinal specialists (R.D.A and M.G.).

**Figure 1:**
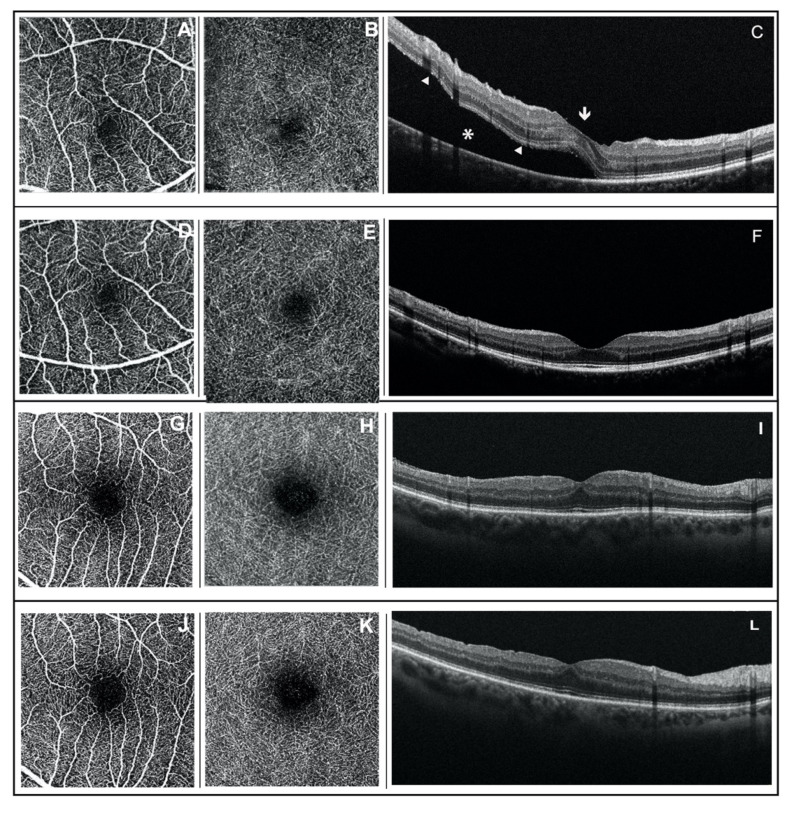
Foveal avascular zone area analysis on optical coherence tomography angiography en-face 3 × 3 images and optical coherence tomography B-Scan in bisected macula rhegmatogenous retinal detachment eye (**A**–**F**) and not bisected macula rhegmatogenous retinal detachment eye (**G**–**L**). (**A**,**G**) Superficial capillary plexus at preoperative time; (**B**,**H**) deep capillary plexus at preoperative time; (**C**,**I**) preoperative OCT B-scan of bisected and not bisected macula retinal detachment, respectively. In the bisected macula group, the subretinal fluid (*) determines a separation of the neurosensory retina (white arrowhead) from the retinal pigment epithelium that transects the fovea (white arrow), despite that, the fovea remains morphologically intact. (**D**,**J**) Superficial capillary plexus at postoperative time; (**E**,**K**) deep capillary plexus at postoperative time; (**F**,**L**) postoperative OCT B-scan of bisected and not bisected macula retinal detachment. In the bisected macula group, restoration of normal morphology has occurred following retinal detachment repair.

The same two ophthalmologists (R.D.A. and M.G.) performed FAZ area calculation by automatically using OCTA integrated system software and by checking and manually delineating the inner edge of the foveal capillaries if errors were detected (Figure 1).

**Figure 2:**
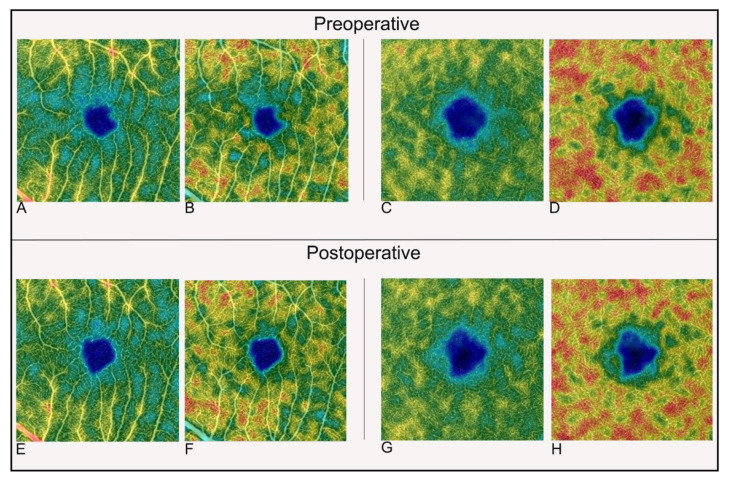
Optical coherence tomography angiography 3 × 3 density map images in macula ON rhegmatogenous retinal detachment eyes. Preoperative vessel density and vessel length density of superficial capillary plexus (**A**,**B**); vessel density and vessel length density of deep capillary plexus at preoperative time (**C**,**D**). Postoperative vessel density and vessel length density of superficial capillary plexus (**E**,**F**); Postoperative vessel density and vessel length density of deep capillary plexus (**G**,**H**).

VD and vessel length density (VLD) were recorded for central area (fVD/fVLD, the area under a circumference of 1 mm diameter around the fovea), parafoveal area (pfVD/pfVLD, annular area extending between 1 and 2.5 mm diameter, centered on the foveola) and whole macular area (wVD/wVLD, area under a circumference of 2.5 mm diameter around the fovea). In addition, parafoveal area was segmented in four quadrants (superior, nasal, inferior and temporal), then VD and VLD were recorded for each of them. All these processes of segmentation of macular OCTA images were performed by integrated images processing system software (Figure 2).

VD and VLD values were calculated, automatically, by the same integrated system, for each plexus.

In order to calculate VD, the software (OCT-HS100 Angio Expert AX^®^) creates a binary image from an OCTA image and indicates the percentage of white pixels in the region by percent (%). Then, for the calculation of VLD (skeletonization of the image), it transforms the lines of a binary image, created from an OCTA image, into thin lines (1 pixel of thickness) and indicates the value obtained by dividing the sum of the length of the thin lines in the region by the area by “mm^−1^”. CMT was calculated using retinal thickness map of SS-OCT, in Macula 3D mode. In the case of BM group RRDs, manual editing of the retinal thickness boundary lines was performed to calculate the CMT value.

### 2.3. Surgical Procedure

All surgical procedures were performed by the same expert vitreoretinal surgeon (R.M.) within 24 hours. All patients were treated with 25 gauge-pars plana vitrectomy, with diluted gas as tamponade (SF6 (20%) or C3F8 (12%)), with final expansible gas injection.

In all cases, standard three-port-PPV was performed with Stellaris vitrectomy machine (Bausch & Lomb Incorporated, Rochester, NY, USA). Central and peripheral vitrectomy was performed, followed by fluid-air exchange and laser retinopexy. Combined cataract surgery was performed in phakic eyes depending on lens status.

### 2.4. Statistical Analysis

Statistical analysis was performed with STATA^®^ software version 14 (StataCorp. 2015. Stata Statistical Software: Release 14. College Station, TX, USA: StataCorp LP). The main outcome parameters included the percentage of BM vs. NBM, and BCVA, CMT, FAZ area of SCP and DCP, VD and VLD of SCP and DCP, expressed as mean ± SD. The normality of the sample distribution was confirmed using the Shapiro–Wilk test (*p* > 0.05). Statistical differences for continuous variable for two groups were examined using paired and unpaired Student’s *t* test. The homogeneity of variances was calculated by Levene Test. The One-Way ANOVA test was used, with post hoc Bonferroni test, to analyze the measurements in each area. Correlation analysis between the values in the different locations were investigated by Pearson’s correlation test. Margins are statistic calculated from predictions of a previously fit model at fixed values of some covariates and averaging. The margins estimate the margins of responses for specified values of covariates and present the results as a figure. 

### 2.5. Box Plot Analysis

Box plots were used to visualize differences in the distribution of numerical data between different groups. The differences between two groups were analyzed by calculating the fold change and *p*-value (Student’s *t*-test).

### 2.6. Scatter Plot Analysis

Scatter plots were used to display relationships between two numeric variables, and the strength and direction of the linear relationships were assessed by Pearson’s correlation coefficient. A *p* < 0.05 was considered statistically significant. *p*-value ≤ 0.05 was considered of statistical significance.

## 3. Results

A total of 14 eyes of 14 patients (eight men, six women) with a mean age of 52.6 ± 15.2 years old were included in this study between November 2020 and April 2021. All patients had a macula ON RRD (six RRD with bisected macula, BM group; eight with not bisected macula, NBM group). The fellow eyes were considered as controls. Patients’ baseline demographic parameters, ocular characteristics and surgical information are summarized in Table 1.

Primary retinal reattachment was obtained in all cases, without recurrences within 3-month follow up. PPV with C3F8 (12%) tamponade was performed in three eyes, while SF6 (20%) was used in 11 eyes. No intraoperative and postoperative complications were observed. None of the eyes included in our study showed extension retinal detachment involving more than two quadrants (Table 1). The retinal detachment mainly involved the superior and temporal sectors (temporal: 78%; superior: 57%, inferior: 28%, nasal: 7%; Table 1).

### 3.1. OCTA Findings

Overall, the mean FAZ area in the affected eyes showed a significant enlargement postsurgery compared to preoperative values, both in the SCP and DCP (*p* = 0.0003 and *p* = 0.0107, respectively) (Table 2).

In a subanalysis between the BM and NBM subgroups, postoperative SCP FAZ enlargement was significant in both groups (Table 3). In detail, for the BM group postoperative SCP FAZ (mean 0.35 ± 0.15 mm^2^) was significantly (*p* < 0.05) improved compared with the preoperative (mean 0.24 ± 0.09 mm^2^) and the fellow group (mean 0.25 ± 0.11 mm^2^). The postoperative SCP FAZ (mean 0.31 ± 0.12 mm^2^) was the largest (*p* < 0.05) compared with the preoperative (mean 0.24 ± 0.08 mm^2^) in NBM group. No significant difference was observed between the preoperative and fellow group in the BM and NBM groups (Table 3).

Conversely, postoperative DCP FAZ significantly increased only in the BM subgroup (Table 3).

**Figure 3:**
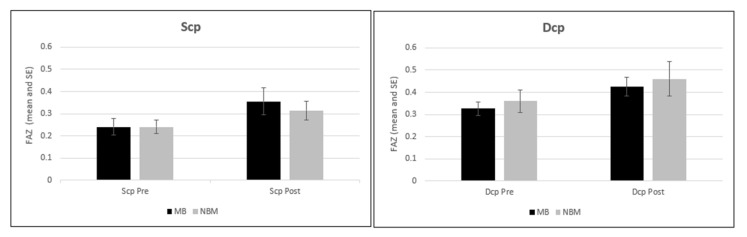
Estimated marginal means preoperative and postoperative described for each DCP and SCP plexuses for the BM and NBM groups.

A significant effect of time was also noted between the two time points (F = 3.68; *p* < 0.017) for FAZ between BM and NBM (Figure 3).

No statistically significant correlations were found between BCVA and FAZ in either the SCP (*p* = 0.50) or DCP (*p* = 0.14). No significant correlations were found between the final BCVA and CMT (*p* = 0.43). An inverse correlation was found between the SCP FAZ and the CMT (*p* = 0.0078) but not for the DCP FAZ (*p* = 0.088).

**Figure 4:**
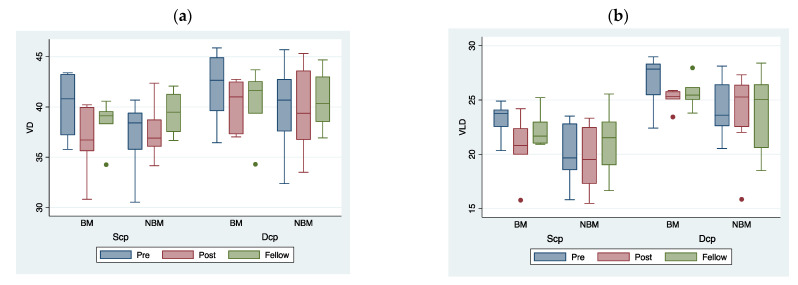
Boxplot showing distribution of VD (**a**) and VLD (**b**) in both DCP and SCP after, before surgery and fellow eye results for the BM and NBM groups. Boxes represent interquartile range, whiskers represent variability and horizontal bars represent the median value.

Regarding perfusion parameters, a decreasing trend was observed in terms of whole macular VD in both the DCP and SCP after surgery. In detail, the quantitative analysis of the individual quadrants showed a trend of decreased perfusion in all sectors except for the nasal one, but not significantly, and the inferior one, which was found to be statistically significant higher in the DCP (*p* = 0.036, Figure 4) and slightly increased in the SCP layer.

An inverse trend between the mean FAZ area, and the values of fVD and fVLD were found both in the SCP and DCP. No correlation was found between the perfusion parameters and final visual outcomes, in both plexuses.

No correlation between the surgery time and postoperative SCP FAZ (*p* = 0.59), DCP FAZ (*p* = 0.69) and CMT (*p* = 0.72) was assessed.

### 3.2. Visual Acuity

No difference in terms of visual acuity was found between RRD eyes and the fellow ones (Table 2). In diseased eyes, the mean BCVA was 0.11 ± 0.20 logMAR and 0.089 ± 0.180 logMAR preoperatively and postoperatively, respectively, without statistical differences (Table 2). The choice of the tamponade gas or the entity of laser photocoagulation (360° or sectorial) had no statistically significant effect on the final visual outcome.

## 4. Discussion

In this prospective observational study, our sample showed changes in retinal architecture and vascularization in patients with early onset macula-ON RRD treated with prompt vitreoretinal surgery (within 24 hours). These early anatomical and perfusion modifications were analyzed using OCTA, which nowadays has become a clinical practice tool able of rapidly and non-invasively assessing macular perfusion and architecture.

At baseline, no significant difference was found between eyes with RRD and fellow eyes in terms of FAZ area, thus suggesting that probably more time is required for anatomical microstructural changes to occur in early onset macula on RRD and that surgery itself may be partially responsible for the structural modification. Indeed, similarly to previous studies, our findings described a statistically significant enlargement of the FAZ area postoperatively, in comparison to preoperative values, in both of the two retinal capillary plexuses (SCP and DCP), as an indirect sign of ischemic changes or retinal manipulation during surgery [17,18,19].

Of note, in our subanalysis between BM and NBM subgroups, postoperative SCP FAZ enlargement was significant in both BM and NBM, while postoperative DCP FAZ enlargement was significant only in the BM subgroup. The maintenance of the foveal microstructure has been strictly linked to the postsurgical visual outcome in macula off retinal detachment. Our data, based on a relatively short follow-up time, reported an excellent visual outcome preservation, given that the macula was not or only partially involved. Nevertheless, in the macula ON RRD condition, some previous works did not report any significant difference between pre and postoperative FAZ values in the eyes of patients who underwent vitreoretinal surgery [17,20,21,22,23,24]. Barca et al. observed a reduction, not statistically significant, in the FAZ area of operated eyes, but this could probably be explained by peeling of the ILM, performed for all patients in the study, which could be responsible for a decrease in and/or distortion of the FAZ area [24,25]. Barca et al. also reported that eyes with macula ON RRD showed significant preoperative lowering of VD when compared with fellow eyes at the SCP level, recovering over time after 6 month follow-up [20,21,24]. The authors explained the phenomenon assuming that peripheral retinal vascular resistance during RRD could induce a detectable slowing of blood perfusion in the SCP. Previous studies on eyes with macula OFF RRD, using fluorescein angiography, had already identified how increased vascular resistance in the detached retina could lead to a reduction in and slowing of blood flow [15,16,26,27]. By means of scanning laser Doppler flowmetry, Eshita T. et al. reported the mechanical effect of scleral buckle indentation on peripheral perfusion, which caused a blood flow decrease just two weeks after surgery in patients with RRD without macular involvement; nevertheless, a complete reperfusion was observed after 1-month follow up, reaching baseline values [27]. Chua et al., in a prospective study reporting 1-year follow-up findings, observed a FAZ area decrease, explained as a likely physiological variability in the FAZ size; nevertheless, they considered only macula OFF RRD patients [28].

Conversely, in our cohort of patients with RRD, at baseline, whole macular and parafoveal VD and VLD of diseased eyes were slightly decreased when compared with the fellow ones, but not significantly. After surgery, the whole macular and parafoveal VD and VLD of our sample had a downward trend in both retinal plexuses. In detail, we noticed a slight decrease in VD and VLD in all retinal layers analyzed (SCP and DCP) and a statistical rise in VD at the deep level of the inferior quadrants of the posterior pole, likely due to the main topographical involvement of retinal detachment in our cohort of patients (43% superotemporal involvement). Yi et al. found that, in the case of hypoxia, an abnormal oxygen metabolism could increase the metabolic demands of the retinal circulation, rather than increasing the extraction of oxygen from the choriocapillaris. Therefore, in the case of RRD, hypoxia would occur in the detached retina with a consequent increased oxygen extraction starting from the retinal vascularization [29]. Moreover, blood flow changes after surgery have been significantly correlated with the extent of RRD [27].

Our findings partially agree with previous publications, where they did not report any changes in VD after vitreoretinal surgery [17,20,21,22,23]. Obviously, it should be taken into consideration that a part of our sample consisted of eyes with macula bisected solved within 24 hours from the onset of symptoms, allowing us to speculate that the retinal perfusion might start to behave as a macula off retinal detachment unless an immediate surgical correction is performed. We may further hypothesize that the retina attached immediately adjacent to the detached retina is more sensitive to hypoxic damage related to slowing blood flow. Despite the retinal flow decreased in retinal detachment pathogenesis, we may assume that this was not sufficiently low or more time was needed from the onset of the retinal detachment to induce microstructural changes detectable with OCTA on the whole macular area in our study population.

Similarly to our results, Yoshikawa et al. reported no significant difference in macular perfusion density between eyes with retinal detachment and the healthy fellow eyes, underlining that early onset retinal detachment keeps the retinal vasculature intact. Differently to this, Bonfiglio et al. focused their attention on a retrospective cohort of patients with an anatomically attached retina after at least 12 months from surgery, dividing them into two subgroups, macula on and off. In the macula on group, no significant difference in postoperative mean FMT, FAZ and SCP VD was found, while a lower mean DCP VD in the parafoveal subfield was assessed. It is still debated as to which capillary plexus is first involved in retinal detachment. Woo et al. hypothesized that the DCP is more vulnerable to a lack of oxygen because of its different anatomical vascular supply and different intraretinal location compared with the SCP, which is directly connected to the retinal arterioles instead of the venous channels. Conversely, other works found that in early onset retinal detachment, especially when the macula is spared, the most affected capillary plexus seems to be the SCP, which is the first vascular layer involved in a fast increase in vascular resistance induced by the RRD. The SCP may develop a stronger and faster contraction than the DCP due to its greater density of arterioles and smooth muscle. Conversely, the DCP, although more vulnerable to damage caused by hypoxia, could be involved later [24].

Moreover, in the retinal detachment pathophysiology, Muller cells seem to be another important factor in the mechanisms of vasoconstriction and hypoxia. Activated by the release of Endothelin-1, they can cause alterations in the internal retinal flow, even in the absence of structural alterations [30,31,32]. In addition, Endotelin-1, which was found to be increased in the subretinal fluid in RRD eyes, has an intrinsic vasoconstrictive action, leading to a reduction in microvascular blood flow [33,34]. Unfortunately, we did not analyze Endothelin-1 or other cytokines’ levels in subretinal fluid.

The unique angle of this prospective work was the analysis of early microvascular and microarchitectural alterations in patients with early onset macula ON retinal detachment who underwent prompt vitreoretinal surgery within 24 hours, thus suggesting a predictive role of such perfusion changes as potential biomarkers of final prognosis after surgery and underlining the importance of a correct timing in macula ON DRR surgery. A subanalysis between bisected and not bisected macula was reported as well, adding a new understanding into the field.

It is still controversial if macular vessel changes could actually be related to visual prognosis. In our study, no statistically significant differences were identified in terms of mean BCVA either between preoperative and postoperative values. This is important given that our study involved only patients with macula ON RRD. In the literature, macular involvement is one of the main factors influencing postoperative visual acuity, especially in relation to the duration of detachment [35,36]. As expected, our sample showed a worse visual acuity in BM patients in comparison with the NBM group. Despite this, no significant differences in final postsurgical BCVA were detected between the two subgroups. This is in accordance with previous studies, where the progression of detachment to macular involvement does not consistently influence visual functional recovery if surgery is performed very quickly (within 24 hours) [26,37,38]. No significant correlation was found between BCVA and FAZ in both the SCP and DCP as well.

In addition to this, in our sample, the inverse relationship between FAZ and CMT was respected as previously reported in several studies, both in healthy and RRD macula ON eyes [15,22,24,39].

## 5. Conclusions

In conclusion, our findings suggest that BM and NBM groups could behave differently in terms of structural parameters such as FAZ. On the other hand, macular perfusion of eyes with macula-on RRD, also with the macula not yet completely involved as in the bisected one, is still preserved if the surgery is performed promptly; thus highlighting the great importance of a correct timing for surgery, in both cases of BM and NBM.

Undoubtedly, our study has some limitations. First, the sample size of the study group was relatively small with a relatively short follow-up period of 3 months. Second, some surgical parameters that could influence the recovery of the capillary plexuses such as the elevation of IOP during surgery were not considered in our study. Third, vitreous or SRF chemokine/cytokines levels to be correlated with perfusion/architectural parameters were not investigated.

Further investigations are needed with a wider sample of patients, with a longer follow-up period and with a correlation between perfusion parameters and the extent of detached retina, to better understand the pathophysiological mechanism underneath these early vascular microarchitecture modifications of the posterior pole in retinal detachment not completely involving the fovea. A prospective longer study would be warranted to confirm and validate this preliminary results.

## Figures and Tables

**Table 1:** Demographics, ocular characteristics and surgical techniques data.

		Patients (n = 14)
Mean Age (Years)		52.6 *±* 15.2
	nNBM	48.13 ± 17.36
	nBM	58.67 ± 10.01
Male: female (n)		8:6
RE: LE (n)		7:7
	NBM	4:4
	BM	3:3
PPV (SF6: C3F8) (n)		11:3
NBM: BM (n)		8:6
Diabetes mellitus ^†^ (n, %)		2 (14.3%)
	nNBM (%)	1 (12.5%)
	nBM (%)	1 (16.7%)
High blood pressure^†^ (n, %)		5 (35.7%)
	nNBM (%)	3 (37.5%)
	nBM (%)	2 (33.3%)
Axial length (mm)		24.8 ± 1.1
	nNBM	25.5 ± 1.0
	nBM	24.3 ± 1.1
Phakic (n)		13
NBM: BM (n)		7:6
Pseudophakic (n)		1
NBM: BM (n)		1:0
PPV + PHACO + IOL % (n)		54.5% (6/11)
NBM: BM (n)		3:3
Surgery Duration (min)		85.36 ± 24.40
	NBM	93.75 ± 24.07
	BM	74.17 ± 21.78
360° laser photocoagulation % (n)		21.43% (3/14)
	NBM	25% (2/8)
	BM	16.67% (1/6)
Detachment extension % (n)		
S (only)		7% (1)
NBM/BM		1/0
S-N		7% (1)
NBM/BM		1/0
N (only)		0% (0)
N-I		0% (0)
I (only)		7% (1)
NBM/BM		1/0
I-T		21% (3)
NBM/BM		0/3
T (only)		14% (2)
NBM/BM		0/2
T-S		43% (6)
NBM/BM		5/1

Abbreviations: n, number of patients; RE, right eye; LE, left eye; PPV, pars plana vitrectomy; SF6, sulfur hexafluoride; C3F8 octafluoropropane; PHACO + IOL, phacoemulsification + intraocular lens implantation; BCVA, best-corrected visual acuity; S, superior; N, nasal; I, inferior; T, temporal; NBM, not bisected macula; BM, bisected macula. ^†^ Defined as ongoing medical treatment at the time of investigation.

**Table 2:** Best-corrected visual acuity, optical coherence tomography and optical coherence tomography angiography mean values.

		Preoperative	Postoperative	Fellow Eye	*p*-Value	
					Pre. vs. Post.	Pre. vs. Fellow
**BCVA** (logMAR)		0.114 ± 0.2	0.089 ± 0.184	0.016 ± 0.059	0.3644	0.1144
	(Snellen)	20/26	20/25	20/20		
**CMT** (µm)		307.50 ± 35.56	302.64 ± 33.84	284.64 ± 26.47	0.5914	**0.0067**
**FAZ** (mm2)						
Scp		0.24 ± 0.08	0.33 ± 0.13	0.24 ± 0.09	**0.0003**	0.8739
Dcp		0.35 ± 0.16	0.45 ± 0.24	0.36 ± 0.15	**0.0107**	0.0941
**Central Area**						
VD (%) Scp		32.24 ± 4.55	29.54 ± 4.79	30.62 ± 4.35	0.1298	0.0758
VD (%) Dcp		32.63 ± 9.81	30.70 ± 6.77	29.63 ± 6.83	0.4378	0.1486
VLD (mm^−1^) Scp		18.22 ± 3.11	16.29 ± 2.72	17.6 ± 2.60	0.1074	0.3980
VLD (mm^−1^) Dcp		19.67 ± 6.35	18.44 ± 4.27	17.77 ± 4.86	0.4443	0.1773
**Quadrant I**						
VD (%) Scp		41.58 ± 3.93	42.01 ± 2.15	42.06 ± 3.45	0.6224	0.7051
VD (%) Dcp		42.72 ± 3.69	44.1 ± 2.42	43.96 ± 3.4	**0.0357**	0.2991
VLD (mm^−1^) Scp		22.94 ± 3.15	22.07 ± 2.89	22.99 ± 3.25	0.2841	0.9500
VLD (mm^−1^) Dcp		26.82 ± 3.12	26.91 ± 2.76	26.47 ± 3.19	0.9294	0.6561
**Quadrant N**						
VD (%) Scp		37.3 ± 7.07	37.34 ± 3.77	39.27 ± 2.42	0.9838	0.3395
VD (%) Dcp		41.51 ± 4.16	41.03 ± 3.70	43.06 ± 2.44	0.7448	0.1063
VLD (mm^−1^) Scp		21.13 ± 4.89	20.54 ± 3.55	21.95 ± 2.73	0.6137	0.4177
VLD (mm^−1^) Dcp		25.63 ± 3.26	25.19 ± 2.77	26.28 ± 2.36	0.6562	0.3093
**Quadrant S**						
VD (%) Scp		41.29 ± 3.90	39.04 ± 4.76	42.84 ± 2.15	0.1884	0.2025
VD (%) Dcp		44.59 ± 2.68	41.88 ± 5.2	43.67 ± 2.53	0.0748	0.1279
VLD (mm^−1^) Scp		22.74 ± 3.22	20.91 ± 3.92	23.34 ± 3.28	0.0779	0.2915
VLD (mm^−1^) Dcp		27.93 ± 2.92	25.84 ± 4.27	26.79 ± 3.56	0.1959	0.2436
**Quadrant T**						
VD (%) Scp		40.34 ± 4.13	37.78 ± 2.72	40.3 ± 1.78	0.0749	0.9774
VD (%) Dcp		42.96 ± 2.96	42.24 ± 2.3	42.75 ± 1.57	0.4299	0.8184
VLD (mm^−1^) Scp		22.41 ± 2.78	20.69 ± 2.95	22.29 ± 2.47	0.1130	0.8921
VLD (mm^−1^) Dcp		26.68 ± 2.75	25.91 ± 3.05	25.89 ± 2.65	0.5471	0.3824
**Whole Macular Area**						
VD (%) Scp		38.55 ± 3.50	37.14 ± 2.88	39.02 ± 2.10	0.2855	0.5915
VD (%) Dcp		40.89 ± 4.00	39.99 ± 3.47	40.61 ± 2.92	0.3868	0.7796
VLD (mm^−1^) Scp		21.49 ± 2.72	20.1 ± 2.88	21.63 ± 2.50	0.0980	0.7796
VLD (mm^−1^) Dcp		25.35 ± 2.76	24.46 ± 2.85	24.64 ± 2.98	0.3633	0.2758
**Parafoveal Macular Area**						
VD (%) Scp		40.13 ± 3.82	39.04 ± 2.88	41.12 ± 1.81	0.3981	0.3580
VD (%) Dcp		42.94 ± 2.77	42.31 ± 2.91	43.36 ± 2.11	0.4068	0.5140
VLD (mm^−1^) Scp		22.31 ± 2.92	21.05 ± 3.19	22.64 ± 2.70	0.1231	0.5150
VLD (mm^−1^) Dcp		26.76 ± 2.55	25.97 ± 2.89	26.36 ± 2.80	0.4472	0.5205

BCVA: Best-corrected visual acuity; CMT: central macular thickness; FAZ: foveal avascular zone; VD: vessel density; VLD: vessel length density; SCP: superficial capillary plexus; DCP: deep capillary plexus.

**Table 3:** Best-corrected visual acuity and optical coherence tomography angiography mean values for the BM and NBM groups.

	BM			NBM		
	Preoperative	Postoperative	Fellow Eye	Preoperative	Postoperative	Fellow Eye
BCVA (logMAR)	0.24 ± 0.26	0.16 ± 0.27	0.04 ± 0.08	0.01 ± 0.03 ^#^	0.03 ± 0.03	0.0 ± 0.0
FAZ (mm^2^)						
Scp	0.24 ± 0.09	0.35 ± 0.15 *	0.25 ± 0.11	0.24 ± 0.08	0.31 ± 0.12 °	0.23 ± 0.07
Dcp	0.32 ± 0.18	0.42 ± 0.29 *	0.34 ± 0.16	0.36 ± 0.14	0.46 ± 0.22	0.37 ± 0.15
Whole Macular Area						
VD (%) Scp	40.21 ± 3.19	36.67 ± 3.43	38.49 ± 2.21	37.30 ± 3.36	37.49 ± 2.58	39.41 ± 2.05
VD (%) Dcp	42.03 ± 3.78	40.26 ± 2.51	40.52 ± 3.38	40.02 ± 4.17	39.78 ± 4.20	40.67 ± 2.76
VLD (mm^−1^) Scp	23.23 ± 1.62	20.66 ± 2.84	22.25 ± 1.66	20.18 ± 2.70	19.67 ± 3.01	21.16 ± 3.01
VLD (mm^−1^) Dcp	26.81 ± 2.46	25.14 ± 0.89	25.64 ± 1.40	24.24 ± 2.56	23.95 ± 3.72	23.88 ± 2.63

* Statistically significant, *p*-value < 0.05 for Faz in BM group, pre vs. post; ° statistically significant, *p*-value < 0.05 for Faz in NBM group, pre vs. post; ^#^ statistically significant, *p*-value < 0.05 for BCVA in preoperative group, BM vs. NBM.

## Data Availability

All data will be available on request to the corresponding author.
